# Comparison of the Efficacy of Percutaneous Microwave Ablation Therapy versus Laparoscopic Partial Nephrectomy for Early-Stage Renal Tumors

**DOI:** 10.3390/diagnostics14141574

**Published:** 2024-07-20

**Authors:** Osman Kula, Yeliz Ateş, Hakkı Mete Çek, Atınç Tozsin, Burak Günay, Burak Akgül, Selçuk Korkmaz, Gökhan Karataş, Serdar Solak, Fethi Emre Ustabaşıoğlu, Ersan Arda

**Affiliations:** 1 Department of Radiology, Faculty of Medicine, Trakya University, Edirne 22030, Turkey; drosmankula@gmail.com (O.K.); gokhankaratas1984@gmail.com (G.K.); serdarsolak@gmail.com (S.S.); ustabasioglu@hotmail.com (F.E.U.); 2 Department of Urology, Private Ekol Hospital, Edirne 22030, Turkey; metecek@gmail.com; 3 Department of Urology, Faculty of Medicine, Trakya University, Edirne 22030, Turkey; atinctozsin@gmail.com (A.T.); burakakgul11@windowslive.com (B.A.); ersanarda@yahoo.com (E.A.); 4 Department of Radiology, Kırklareli Research and Training Hospital, Kırklareli 39010, Turkey; drburakgunay@gmail.com; 5 Department of Biostatistics, Faculty of Medicine, Trakya University, Edirne 22030, Turkey; selcukorkmaz@gmail.com

**Keywords:** laparoscopic partial nephrectomy, percutaneous microwave ablation therapy, renal cell carcinoma

## Abstract

This study aimed to compare the efficacy of percutaneous microwave ablation therapy (MWAT) and laparoscopic partial nephrectomy (LPN) in early-stage renal cell carcinoma (RCC) classified as T1a; a retrospective analysis was conducted on patients treated between January 2017 and November 2023. Oncological outcomes, radiological recurrence, length of stay (LOS), and costs were evaluated. The study included 110 patients, with no significant differences between the two groups regarding residual tumors, local tumor progression, and disease-free survival rates (*p* > 0.05). The LPN group showed significantly lower pre/postoperative serum urea and creatinine and higher estimated glomerular filtration rate values, whereas the MWA group experienced significantly lower mean costs, complication rates, LOS in the hospital, and procedure durations (*p* ≤ 0.05). However, post-procedure residual tumors and local tumor progression rates did not differ significantly between the LPN and MWAT groups (*p* > 0.05). MWAT is as effective as LPN for T1a RCC lesions. In addition, MWAT has lower costs than LPN and is a cost-effective treatment method. Therefore, MWAT minimizes hospital stay and complications and since the oncological results are similar to LPN, it might be considered as the first choice of treatment in young patients.

## 1. Introduction

The wider use of imaging modalities has allowed the increased detection of incidental renal cell carcinoma (RCC) [1]. Approximately 60% of RCCs are diagnosed incidentally via cross-sectional imaging. Hence, life expectancy has substantially increased for these patients [2]. Early-stage RCC is known as stage T1a, defined as a non-invasive mass of less than 4 cm in the kidney.

Current treatment modalities of RCC include radical and partial nephrectomy, active surveillance (AS), radiofrequency ablation (RFA), cryoablation (CA), and percutaneous microwave ablation therapy (MWAT) [3,4]. Treatments should be selected on an individual basis, considering the estimated residual kidney function, the possibility of developing chronic kidney disease, long-term survival, possible recurrence, and residual potential [5]. One of the preferred surgical options is nephron-sparing nephrectomy because the renal function is preserved, and the procedure has low complication rates compared to total/radical nephrectomy [6]. However, in patients with high comorbidity, small changes in renal function after surgery may lead to cardiovascular problems and increase mortality [7]. Thus, percutaneous thermal ablation treatments, which are considered safer and can minimize surgical risks and potential postoperative complications, are widely used to treat T1a RCC patients. This approach is particularly advantageous when patient comorbidities are considered [8]. Recent studies have shown that MWAT can be used more frequently in fragile patient groups [9].

Percutaneous treatments hampered the progression of chronic renal failure [7,10]. Percutaneous ablative procedures such as RFA and CA exhibited similar results [11,12]. Many studies have shown that RFA and CA perform equally well regarding recurrence or residual tumors, based on follow-up radiological imaging [13]. MWAT has become popular recently because of its reliability and efficacy [14,15]. MWAT is widely used because of its advantages, including percutaneous application, high-heat technology, ability to perform wider ablation, and antitumoral immune modulation [14]. MWAT can ablate a larger zone than RFA and is more potent in completely eradicating tumors [4,16].

Thus, MWAT should be considered an alternative to laparoscopic partial nephrectomy (LPN) in T1a RCC patients who cannot undergo general anesthesia because of comorbidities, diagnosed with chronic kidney disease, and in those with hereditary RCC lesions due to von Hippel–Lindau (VHL) syndrome, bilateral RCC, and solitary kidneys [1]. MWAT can also be part of the initial treatment for patients unwilling to undergo major surgery and those technically unsuitable for LPN.

## 2. Materials and Methods

**I. Study Design and Subjects**

**Figure 1:**
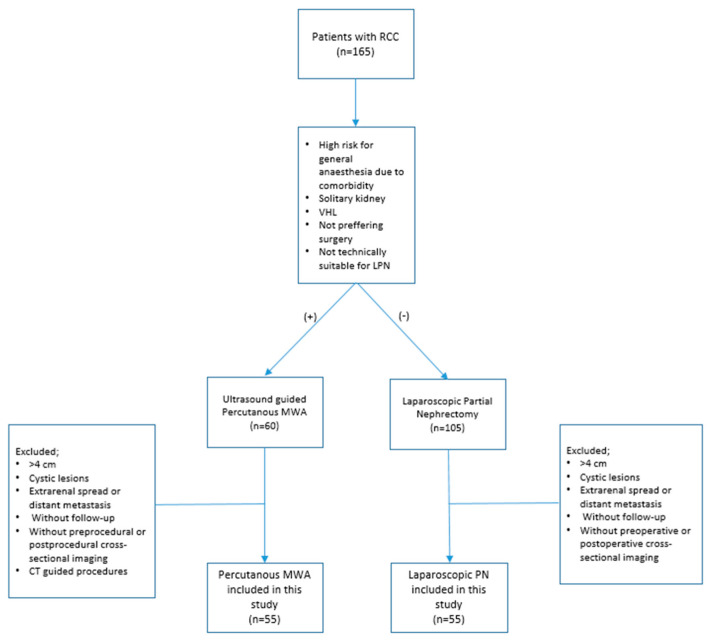
Patient flowchart. PN = partial nephrectomy, MWA = microwave ablation, RCC = renal cell carcinoma.

The local ethics committee approved (TÜTF GOBAEK 2023/507) and written informed consent was acquired from patients. A total of 110 patients diagnosed with T1a RCC, who underwent either LPN or percutaneous MWAT between January 2017 and November 2023, were retrospectively analyzed. The patients’ medical records were obtained from the hospital’s electronic data system. The study considered solid lesions of less than 4 cm, histopathologically diagnosed as RCC, and classified as T1a. The study excluded patients with lesions larger than 4 cm, cystic lesions, extrarenal spread, or distant metastasis at diagnosis. Furthermore, patients who did not undergo cross-sectional imaging before diagnosis and those who received computed tomography (CT)-guided MWAT were excluded. Patients who did not comply with the follow-up were also excluded from the study. Figure 1 shows the inclusion and exclusion criteria.

**Figure 2:**
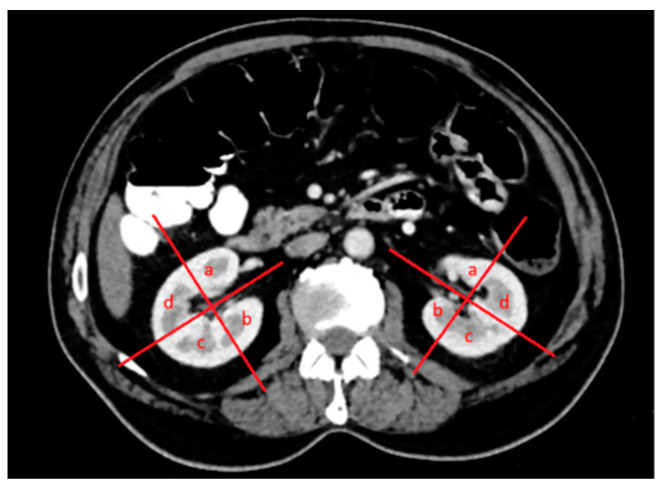
Both kidneys in the axial venous-phase computed tomography (CT) image were divided into four quadrants in the upper, middle, and lower poles, with a line drawn parallel to the vascular structures originating from the hilus-collecting system and a second line perpendicular to it: (**a**) anteromedial, (**b**) posteromedial, (**c**) posterolateral, and (**d**) anterolateral.

**Figure 3:**
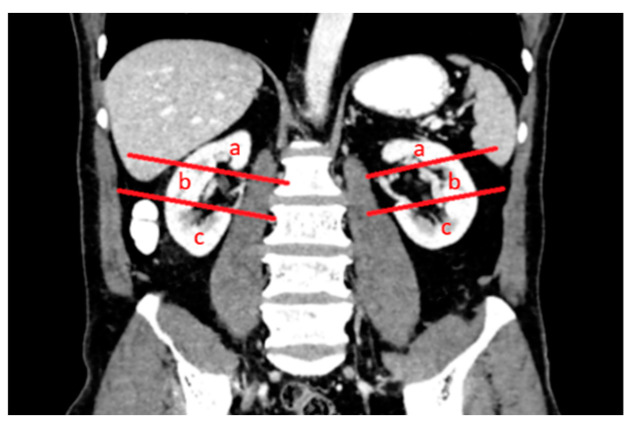
Polar lines (red lines) parallel to the hilar vascular structures of both kidneys were identified in the coronal venous-phase CT image. The upper, middle, and lower pole locations of the lesions were determined according to the upper and lower polar lines and the location of the lesions was according to the L component of the R.E.N.A.L. nephrometry scoring: (**a**) upper pole, (**b**) middle pole and (**c**) lower pole.

**Figure 4:**
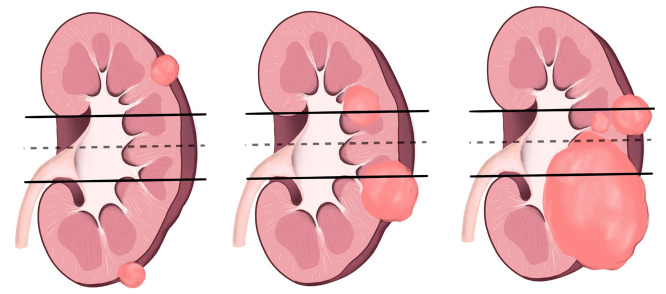
L component of R.E.N.A.L. nephrometry scoring is shown in the illustration above.

The patients’ demographic characteristics, diagnosis of a solitary kidney, diagnosis of chronic kidney disease, diagnosis of the von Hippel–Lindau (VHL) syndrome, previous kidney surgery history, size and location of renal lesions, relationship to pole lines and parenchyma, location within the kidney, and distance from the hilus were analyzed (Figure 2, Figure 3 and Figure 4). Complications were assessed and classified, and data on length of stay (LOS), surgery duration, costs, and serum urea and creatinine levels before and after surgery were obtained from the hospital records. Estimated glomerular filtration rate (e GFR) values were calculated in both groups before and after the procedure. Follow-up data included the presence of residual tumors or local tumor progression after the procedure.

**II. Pre-procedural Imaging and Patient Evaluation**

All patients underwent ultrasound (US) with a 3.5–5.0 MHz convex multifrequency transducer and Aplio 500 Platinum US device (Canon Medical Systems, Otawara, Japan) before the procedure. The position given to the patient during the procedure (prone or lateral decubitus) was determined by lesion location. Percutaneous MWAT was performed under US guidance in all patients. After the procedure, bleeding and hematoma were screened with US. All patients were diagnosed using cross-sectional methods, primarily computed tomography (CT) or magnetic resonance imaging (MRI). Dynamic contrast-enhanced upper abdominal MRI was the preferred imaging method. However, patients unable to undergo MRI due to obesity, claustrophobia, contrast material allergy, metal implants, or metallic foreign bodies were evaluated with intravenous (IV) contrast-enhanced CT instead.

**III. Treatments**

The treatment decision was made by team consensus; the team comprised an interventional radiologist (O.K.) and two urologists (E.A. and H.M.Ç.) with more than 10 years of experience in the treatment of RCC. MWAT was performed in patients with solitary kidneys, patients at high risk for general anesthesia, patients who refused surgery, those who were technically unsuitable for LPN due to lesions in the proximity of the collecting duct and hilus, and those with VHL syndrome. LPN was performed in patients who were suitable for LPN.

**Figure 5:**
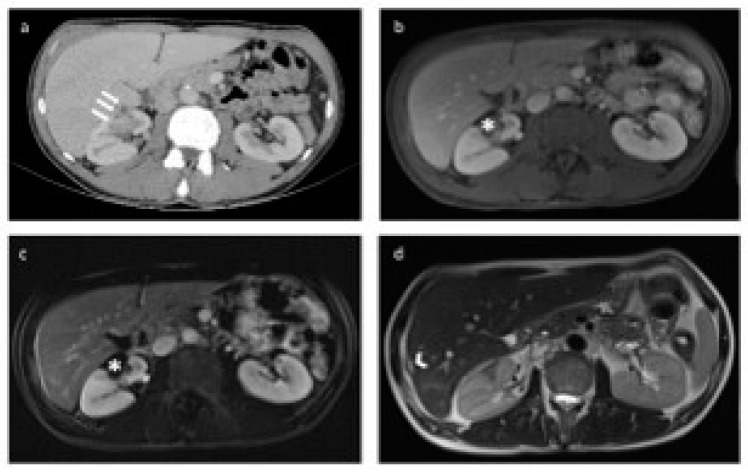
Preoperative computed tomography (CT) and postoperative magnetic resonance (MR) images of the patient treated with percutaneous microwave ablation therapy (MWAT) via transhepatic approach. (**a**) In the axial-plane postcontrast portal venous phase CT image, a lesion (white arrow) is observed in the anteromedial aspect of the middle pole of the right kidney before the operation, in close proximity to the renal vascular structures. (**b**,**c**) No residual lesion was detected in the tumor bed (asterisk) after percutaneous MWAT on axial postcontrast portal venous phase and subtracted T1-weighted MR images. (**d**) In the axial-plane T2-weighted MR image, the loculation is observed in the neighborhood of the liver segment 7-6 junction, which is compatible with the transhepatic ablation trace (arrowhead).

**Figure 6:**
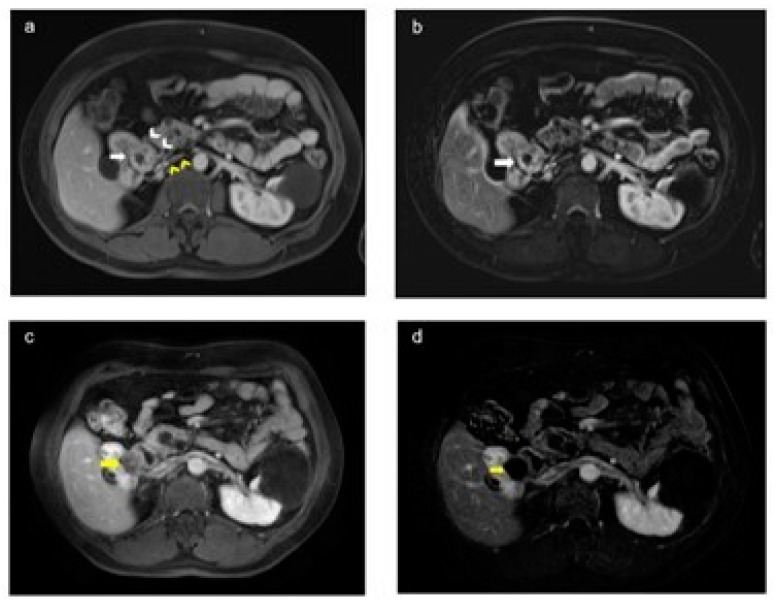
Preoperative and postoperative magnetic resonance (MR) images in a patient with von Hippel–Lindau (VHL) syndrome (**a**,**b**). The axial plan postcontrast portal venous phase and T1-weighted images show a preoperative lesion (white arrow) in the upper pole of the right kidney, in close proximity to renal vascular structures (yellow arrowhead) and the intestine (white arrowhead) (**c**,**d**). The axial postcontrast portal venous phase and subtracted T1-weighted images show no residual lesion in the tumor bed (yellow arrow) after laparoscopy-assisted ultrasound-guided perioperative microwave ablation therapy.

MWAT was performed according to whether they allowed access by passing through pararenal fat only, by a transrenal approach, or by a transhepatic approach for lesions in the right kidney (Figure 5). Patients in whom the lesion could not be reached by these methods were identified as candidates for laparoscopic US-guided percutaneous MWAT (Figure 6).

### 2.1. Percutaneous Microwave Ablation Therapy

Fifty-two patients were positioned according to the pre-procedural US evaluation. Subsequently, all patients were administered sedoanalgesia by an anesthetist before the procedure. Patients in the percutaneous MWAT group underwent a single biopsy with an automated 18-gauge sharp needle under US guidance before the procedure. MWAT was performed by an interventional radiologist under US guidance, using the appropriate angle and technique. The cool tip needle antenna (Emprint HP Ablation Generator (CAGENHP) operating at 2.45 GHz with a maximum power of 150 W and an Emprint Percutaneous Antenna (CA15L2) with Thermosphere Technology (13 G, 15–20 cm) with increased wave stiffness and perfusion cooling) was used for MWAT. The antenna was placed in the center of the lesion and MWAT was applied at 100 W for 6–10 min, depending on the lesion size, to perform ablation. The procedure was ended after tract ablation.

### 2.2. Laparoscopic Partial Nephrectomy

LPN was performed on 55 patients. Of these, six patients underwent off-clamp LPN and 49 underwent conventional LPN. Patients received general anesthesia and were placed in the lateral decubitus position. A Veress needle was inserted into the abdomen through an incision made at the lateral border of the rectus fascia, over the umbilicus. Intraabdominal pressure was adjusted to 14 mmHg. After placing a 10 mm optical port, two ports (a 5 mm and a 10 mm port) were inserted under direct vision. The transperitoneal cavity was assessed to expose the kidney and tumor. In off-clamp LPN, the renal artery was clamped before tumor removal. The renal capsule was incised near the tumor, which was excised cautiously. Bleeding was managed with bipolar or monopolar coagulation. After complete tumor removal, the tumor bed was sutured using barbed or polyglactin materials and reinforced with Hem-o-Lok clips during renorrhaphy. The specimen was extracted using an organ bag. A Hemovac drain was placed at the operation site.

### 2.3. Laparoscopic Ultrasound-Guided Percutaneous Microwave Ablation Therapy

Cold irrigation was used in three patients during the laparoscopic MWAT procedure to keep the intraoperative temperature within the renal pelvis low. Before placing the patient in the lateral decubitus position, a 19.5 Ch cystoscope was inserted into the bladder through the external urethral meatus in the lithotomy position. The ureteral orifice of the side to be treated was identified, and a guidewire was passed through it. An open-end ureteral catheter was then inserted over the guidewire. After confirming the placement of the ureteral catheter in the renal pelvis by X-ray imaging, the catheter was left in place, and the cystoscope was removed from the urethra. The ureteral catheter was sterilized and brought into the sterile field. Subsequently, the patient was positioned in the lateral decubitus position. The abdomen was accessed using the port arrangement typically employed during LPN. The colon was displaced, exposing the kidney in the retroperitoneal space. Once the area for MWAT was prepared, the tumor was accessed via a percutaneous antenna. Normal saline infusion at room temperature was initiated through the ureteral catheter and irrigation was continued throughout the MWAT.

**IV. Follow-up and Outcomes**

After completion of the percutaneous MWAT, all patients were admitted to the urology clinic of Trakya University. None of them required intensive care unit (ICU) post-procedure. Following the procedure, the MWAT group was observed for a mean of 24 h, while the LPN group was followed up for a mean of 4 days and discharged. The postoperative information, LOS, surgery duration, decrease in hemoglobin, and changes in laboratory values related to the urinary system and renal function were recorded for all patients. The second follow-up was performed at 3 months. Subsequent follow-ups were conducted at 6-month intervals over 2 years. Lesions in the ablation zone or resection bed, detected in the first month by radiological imaging, were evaluated to determine whether residual tumor tissue was present. Newly developed lesions observed in these locations on subsequent imagings were considered local tumor progression. Complications occurring within the first 30 days after surgery were recorded. The Clavien–Dindo classification was used to classify complications (Table 1).

**VI. Statistical Analysis**

Normality was tested using the Shapiro–Wilk test for numerical variables. For group comparisons, the Student’s *t*-test was used if the data followed a normal distribution; otherwise, the Mann–Whitney U test was used. Pearson’s chi-squared or Fisher’s exact tests were used to investigate associations between categorical variables. Kaplan–Meier plots were created, and log-rank tests were performed to compare disease-free survival between both procedures. Numerical variables were presented as the mean and standard deviation if the data followed a normal distribution; otherwise, they were presented as the median with minimum and maximum values. Categorical variables were presented as frequencies and percentages. Statistical significance was set at *p* < 0.05. All analyses were performed using IBM SPSS Statistics 20.

## 3. Results

A total of 110 patients (55 in the LPN group and 55 in the MWAT group) were included in our study. Three patients who underwent laparoscopic US-guided MWAT were included in the MWAT group; 69 patients were male and 41 were female. The mean age was 56 years in the LPN group and 64 years in the MWAT group. Table 2 presents the demographics of the patients. The mean tumor size was 30 mm (min: 10 mm, max: 43 mm) in LPN patients and 23 mm (min: 8 mm, max: 46 mm) in MWAT patients. In the MWAT group, 51% (28) of patients had a mean tumor size between 2 and 3 cm, while in the LPN group, 60% (33) had tumors sized over 3 cm (Table 3). No radiological invasion of the renal calyces or pelvic fat was observed in the patients included in the study. 

Before the procedure, the mean serum urea levels were 33 mg/dL in the LPN group and 38 mg/dL in the MWAT group. Post-procedure urea levels decreased to 27 mg/dL in the LPN group and 33 mg/dL in the MWAT group. Similarly, the mean serum creatinine values before the procedure were 0.80 mg/dL in the LPN group and 0.90 mg/dL in the MWAT group. After the procedure, they were 0.76 mg/dL in the LPN group and 0.93 mg/dL in the MWA group. The mean of preprocedural e GFR values were 78.5 mL/min/1.73 m^2^ in the MWAT group and 98.3 mL/min/1.73 m^2^ in the LPN group, while the mean of post-procedural e GFR values were 77.8 mL/min/1.73 m^2^ in the MWAT group and 98.3 mL/min/1.73 m^2^ in the LPN group. The differences in the serum urea and creatinine levels and e GFR values before and after the procedure were significant (*p* ≤ 0.05). The LPN group showed lower pre/postoperative serum urea, creatinine, and higher e GFR values than the MWA group. Before the procedure, nine patients in the MWAT group were diagnosed with chronic kidney disease (CKD), while four patients in the LPN group were diagnosed with CKD. No progression to end-stage kidney disease or up-grading in CKD was observed after the procedure in both groups. There were no patients with solitary kidneys in the LPN group, whereas four patients (7.3%) in the MWAT group had solitary kidneys. Similarly, there were no patients with previous kidney surgery in the LPN group, whereas three patients were in the MWAT group. While four patients in the MWAT group were diagnosed with VHL syndrome, there were no patients in the LPN group diagnosed with VHL syndrome. Nevertheless, no significant differences were observed between the groups regarding solitary kidney, CKD, previous kidney surgery, and VHL syndrome (*p* > 0.05) (Table 2 and Table 4).

The mean cost of the procedure was USD 1051 (446–1843) for the LPN group and USD 828 (162–1637) for the MWAT group, a significant difference (*p* ≤ 0.05) (Table 4).

The LPN group exhibited an 11% (6/55) complication rate, whereas, for the MWAT group, it was 3.6% (2/55). Complications were classified according to the Clavien–Dindo classification. Two complications were observed in the MWAT group: an abscess treated with antibiotics (Grade 1) and bleeding treated with a transfusion (Grade 2). Complications in the LPN group were distributed as follows: fever managed with antipyretics and hydration in one patient (Grade 1), hemorrhage treated with a blood transfusion in two patients (Grade 2), hemorrhage treated with an endovascular intervention under local anesthesia in two patients (Grade 3a), and hemorrhage treated with surgery under general anesthesia in one patient (Grade 3b). No Grade 4 or 5 complications were observed in any group (Table 4).

The LOS in the hospital was 4 (2–15) days in the LPN group and 1 (1–5) day in the MWA group, a significant difference (*p* ≤ 0.05). The minimum postoperative follow-up was 6 months and the maximum was 71 months, with a mean of 40 months in the LPN group and 13 months in the MWA group. At the mean routine follow-up, 5.5% (3/55) residual disease was observed in the LPN group and 3.6% (2/55), in the MWA group. In addition, one case of local progression was observed in each group. Post-procedure residual tumors and local tumor progression rates did not differ significantly between the LPN and MWA groups (*p* > 0.05). The oncological results are presented in Table 5.

**Figure 7:**
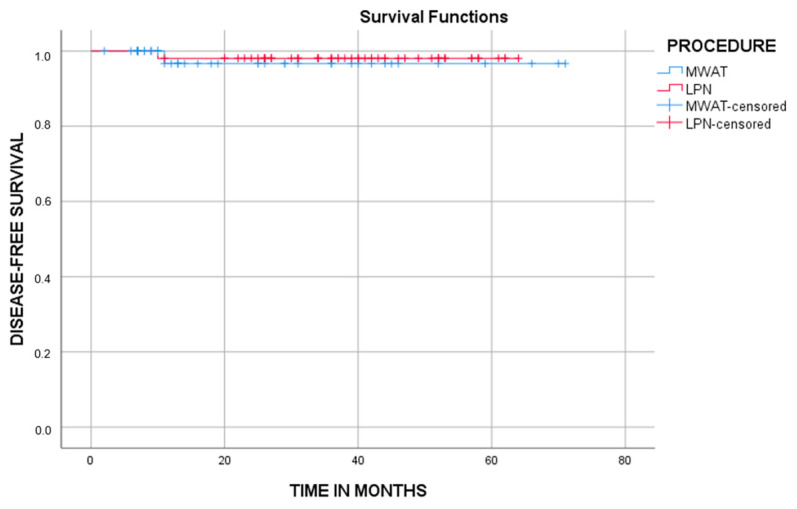
The graph shows cumulative disease-free survival (DFS). There was no significant difference between both groups (*p* = 0.741, log-rank test).

The mean disease-free survival period was 69 (65.1, 72.8) months in the MWAT group and 62.9 (60.9, 64.9) months in the LPN group, and there was no significant difference between both groups (95% confidence interval, *p* = 0.741). Overall survival analysis could not be performed in patient groups due to insufficient follow-up periods (Table 6, Figure 7). Nevertheless, the MWA group had significantly lower mean costs, complication rates, LOS in the hospital, and procedure times (*p* ≤ 0.05).

## 4. Discussion

The mean age of the MWAT group in our study was similar to that in previous studies [11]. The group of patients treated with LPN was younger than the MWAT group, which may be explained by the fewer comorbidities in the former [17].

In several studies, MWAT was found to be markedly less expensive than LPN [14,18]. Percutaneous thermal therapies such as CA and RFA were also reported to be cheaper than open or robotic partial nephrectomy for similar masses [19,20]. Thus, we can assume that the MWAT procedure was cost-effective compared to LPN at rates similar to those reported in the literature.

Studies have shown that, compared to percutaneous ablative procedures, LPN is associated with higher estimated blood loss, operative time, and perioperative complication rates [21,22]. Katsanos et al. found that thermal ablation of small renal masses had similar oncological outcomes to nephrectomy, with notably lower complication rates [10]. The perioperative complication rate in our study was higher in patients who underwent LPN. Three patients in this group experienced major complications requiring interventional procedures under local and general anesthesia. By contrast, there were no complications requiring intervention in the MWAT group.

Furthermore, MWAT was more effective than LPN regarding procedure time and LOS in the hospital. Thus, we can conclude that MWAT is more advantageous regarding patient comfort.

There were no patients with solitary kidneys in the LPN group, whereas four patients (7.3%) in the MWAT group had solitary kidneys. Similarly, there were no patients with VHL syndrome in the LPN group, while there were four patients diagnosed with VHL syndrome. Nevertheless, these differences were not significant (*p* > 0.05). Ablative procedures are recommended for patients with solitary kidneys and hereditary RCC such as VHL syndrome [1]. Our findings may support the preference for MWAT in patients with solitary kidneys or VHL syndrome, agreeing with the literature.

The postoperative assessment includes criteria such as renal function, rates of renal parenchymal disease, and dialysis requirements [23]. Decreased renal function after renal surgery reduces the treatment’s efficacy and is associated with lower patient survival [7,11,23]. Therefore, our study included serum urea and creatinine levels in assessing renal function. Postoperative serum urea and creatinine levels were significantly lower, and the e GFR values were significantly higher in the LPN group than in the MWAT group (Table 4). When considering preoperative renal function tests, differences were significant in both groups, with preoperative serum urea and creatinine values being significantly higher and eGFR values being significantly lower in the MWAT group than in the LPN group (*p* ≤ 0.05). The MWAT group exhibited greater postoperative renal function. This is because patients with high preoperative serum urea and creatinine levels are generally monitored for chronic renal failure or predisposed to renal dysfunction because of comorbidities and, therefore, could not undergo general anesthesia and were treated with MWAT instead of LPN.

Minimally invasive procedures to treat early-stage T1 RCC are increasingly common in uroradiology [5,10,13,24]. Thermal ablative treatments are a step forward for high-comorbidity elderly patients or patients with comorbidities, unable to tolerate surgery [17,25]. MWAT is an increasingly popular high-tech ablative procedure [26]. MWAT is superior to other thermal ablation therapies because it achieves high temperatures quickly, generates a wider and more homogeneous ablation zone, performs the ablation process faster, and is less affected by the physical properties of the ablation site or the cooling effects of circulation. Moreover, MWAT results in adequate results comparable to those of known procedures such as CA regarding safety, preservation of renal function, and oncological efficacy [13,27,28]. Although no quantitative studies provide clear information about the disease-free survival and overall survivability of patients subjected to ablative procedures such as MWAT in recent years, the position of this technique in clinical practice will be clarified by comparing it with other ablative procedures and LPN as the frequency of the procedure increases and case series are established.

A review of the literature shows that recurrence and residual disease rates are slightly higher with MWAT than with LPN [29,30]. This is attributed to the effect of hypervascularization and the lowered effectiveness of thermal ablation when the mass is close to the main vessels and the target temperatures are insufficiently high [29]. Recent systematic reviews and meta-analyses have reported that recurrence-free and cancer-specific survival rates are similar for percutaneous ablative treatments and LPN [10,15,24]. In our study, residual tissue was found in two patients after MWAT and three patients after LPN. The local tumor progression was observed at similar rates in both groups. No significant differences were observed between these methods regarding residual tumors and local tumor progression (*p* > 0.05). Nevertheless, the comparison was skewed because MWAT has been used more frequently in recent years.

Surgery has been defined as the first line of treatment in the guidelines because of the high rates of success of LPN in the treatment of RCC in young and comorbidity-free patients. Surgery has also been defined as a curative treatment [11]. We found no differences between MWAT and LPN concerning residual tumors and local tumor progression. Thus, the success of this procedure in recent years, lower complication rates, and costs suggest that MWAT might be considered as the first choice of treatment in young patients.

Notwithstanding, we could not evaluate overall survival because our study compared procedures over 6 years and MWAT has been widely preferred in recent years. Despite the limitations of our study, we observed that the ablative efficacy of MWAT yields positive outcomes over time. These findings highlight the need for large and comprehensive studies in the future and demonstrate the potential of MWAT as an effective ablation method.

## 5. Limitations

This study had several limitations; it was conducted in a single center and had a retrospective design, which may limit the overall validity and applicability of our results. In addition, the number of patients is limited, which may restrict the study’s statistical power. Some patients had a clinical follow-up of less than 1 year, which affects our survival, metastasis-free, and relapse-free statistics. Thus, assessing long-term outcomes is challenging, which prevents us from fully understanding the true potential of certain diseases. We plan to address these shortcomings in future studies and aim to achieve more comprehensive and reliable results using AI methods. This may allow future studies to include a larger group of patients and longer follow-up data so that we can obtain more precise information on treatment strategies and prognostic assessments.

## 6. Conclusions

Percutaneous microwave ablation therapy (MWAT) shows similar efficacy to laparoscopic partial nephrectomy (LPN) for T1a renal cell carcinoma (RCC) lesions but is cost-effective for patients. Also in MWAT, the length of stay in the hospital is shorter, major complication rates are lower than the LPN, and oncological outcomes are similar in both groups. For this reason, MWAT might be considered as the first-line treatment option and a more comfortable choice for younger patients who are suitable for it.

## Figures and Tables

**Table 1:** Clavien Dindo Classification of Surgical Complications.

Grade	Definition
Grade I	Any deviation from normal postoperative course without the need pharmacological treatment or surgical, endoscopic or radiological interventions. Allowed therapeutic regimens are drugs such as antiemetics, analgesics, diuretics, electrolytes, and physiotherapy. This grade also includes wound infections opened at the bedside.
Grade II	Requiring pharmacological treatment with drugs other than such allowed for Grade I complications. Blood transfusions an total parenteral nutrition are also included.
Grade III	Requiring surgical, endoscopic, or radiological interventions.
Grade IIIa	Intervention not under general anesthesia
Grade IIIb	Intervention under general anesthesia
Grade IV	Life-threatening complications (including CNS complications) * requiring IC-ICU management.
Grade IVa	Single organ dysfunction (including dialysis)
Grade IVb	Multiorgan dysfunction
Grade V	Death of patient
Suffix ‘’d’’	If the patient suffer from a complication at the time of discharge the suffix ‘’d’’ (for ‘’disability’’) is added to the respective grade of complication. This label indicates the need for a follow-up fully evaluate the complication.

* Brain hemorrhage, ischemic stroke, subarachnoid bleeding, but excluding transient ischemic attacks. CNS = central nervous system, IC = intermediate care, ICU = intensive care unit.

**Table 2:** Comparison of Baseline Participant Characteristics between the Percutaneous MWA and LPN Groups.

Parameter	MWA (n = 55)	LPN (n = 55)	*p* Value
Age (year)	64 ± 13	56 ± 13	0.002 *
Sex			0.167
Male	38 (69%)	31 (56%)	
Female	17 (31%)	24 (44%)	
Solitary kidney			0.118
Yes	4 (7.3%)	0 (0%)	
No	51 (93%)	55 (100%)	
Preoperative serum urea (mg/dL)	38 (12, 134)	33 (15, 63)	0.040 *
Preoperative serum creatinine (mg/dL)	0.90 (0.52, 7.42)	0.80 (0.51, 1.80)	0.013 *
Preoperative e GFR (mL/min/1.73 m²)	78.5 (7.8, 118)	98.3 (36.7, 139.3)	0.001 *
Chronic kidney disease			0.067
Yes	9 (16.4%)	3 (5.5%)	
No	46 (83.6%)	52 (94.5%)	
Previous kidney surgery			0.242
Yes	3 (5.5%)	0 (0%)	
No	52 (94.5%)	55 (100%)	
VHL syndrome			0.118
Yes	4 (7.3%)	0 (0%)	
No	51 (92.7%)	55 (100%)	

Data summarized as mean±standard deviation, counts (pencentages) and median (minimum, maximum). MWA = microwave ablation, LPN = laparoscopic partial nephrectomy, e GFR = estimated glomerular filtration rate, VHL = von Hippel Lindau. * Statistically significant difference.

**Table 3:** Comparison of Tumor Characteristics between the Percutaneous MWA and LPN Groups.

Parameter	MWA (n = 55)	LPN (n = 55)	*p* Value
Tumor diameter (mm)	23 (8, 46)	30 (10, 43)	<0.001 *
Tumor diameter subgroups			<0.001 *
≤2 cm	19 (35%)	13 (24%)	
2–3 cm	28 (51%)	9 (16%)	
≥3 cm	8 (15%)	33 (60%)	
Tumor side			0.849
Left	26 (47%)	27 (49%)	
Right	29 (53%)	28 (51%)	
Tumor location on CC axis			0.883
Upper pole	15 (27%)	13 (24%)	
Middle pole	18 (33%)	20 (36%)	
Lower pole	22 (40%)	22 (40%)	
Tumor location on AP axis			0.243
Anterior	19 (35%)	25 (45%)	
Posterior	36 (65%)	30 (55%)	
Tumor location on ML axis			0.003 *
Medial	13 (24%)	28 (51%)	
Lateral	42 (76%)	27 (49%)	
Tumor location relative to parenchyma			0.018 *
Completely endophytic	23 (42%)	10 (18%)	
<50% exophytic	11 (20%)	20 (36%)	
≥50% exophytic	21 (38%)	25 (45%)	
Proximity to renal sinus or collecting system (mm)	3.0 (0.0, 21.0)	4.0 (0.0, 18.0)	0.676
N score			0.922
1	15 (27%)	16 (29%)	
2	7 (13%)	8 (15%)	
3	33 (60%)	31 (56%)	
L score			0.676
1	16 (29%)	14 (25%)	
2	12 (22%)	16 (29%)	
3	27 (49%)	25 (45%)	

Data summarized as counts (pencentages) and median (minimum, maximum). MWA = microwave ablation, LPN = laparoscopic partial nephrectomy, CC (craniocaudal), ML (mediolateral), AP (anteroposterior). * Statistically significant difference.

**Table 4:** Comparison of Intraoperative and Postoperative Outcomes between the Percutaneous MWA and LPN Groups.

Parameter	MWA (n = 55)	LPN (n = 55)	*p* Value
Postoperative serum urea (mg/dL)	35 (14, 119)	27 (8, 73)	<0.001 *
Postoperative serum creatinine (mg/dL)	0.93 (0.52, 8.80)	0.76 (0.50, 1.60)	0.003 *
Postoperative e GFR (mL/min/1.73 m²)	77.8 (6.3, 118)	98.3 (51.8, 133.3)	0.002 *
Procedure time (Min)	32,8(28, 37)	113 (94,177)	<0.001 *
Hospitalization time (Day)	1.00 (1.00, 5.00)	4.00 (2.00, 15.00)	<0.001 *
Complication			0.271
Yes	2 (3.6%)	6 (11%)	
No	53 (96%)	49 (89%)	
No. Of Patients on Clavien-Dindo Classification of Complications			>0.999
Grade 1	1 (50%)	1 (17%)	
Grade 2	1 (50%)	2 (33%)	
Grade 3A	0 (0%)	2 (33%)	
Grade 3B	0 (0%)	1 (17%)	
Cost (U.S. dollars)	828 (162, 1,637)	1,051 (446, 1,843)	0.002 *
Follow-up time (Month)	13 (6, 71)	40 (11, 64)	<0.001 *

Data summarized as counts (pencentages) and median (minimum, maximum). MWA = microwave ablation, LPN = laparoscopic partial nephrectomy, e GFR = estimated glomerular filtration rate. * Statistically significant difference.

**Table 5:** Comparison of Oncologic Outcomes and Recurrence between the Percutaneous MWA and LPN Groups.

Parameter	MWA (n = 55)	LPN (n = 55)	*p* Value
Residual tumoral lesion	2 (3.6%)	3 (5.5%)	>0.999
Local tumor progession	1 (1.8%)	1 (1.8%)	>0.999

Note—Unless otherwise specified, data are numbers of patients, with percentages in parentheses.

**Table 6:** Comparison of Survival Period between the Percutaneous MWA and LPN Groups.

Parameter	MWA	LPN	*p* Value
Disease free survival (month)	69 (65.1, 72.8)	62.9 (60.9, 64.9)	0.741
Overall survival	No exist	No exist	

Data summarized as median (minimum, maximum). MWA = microwave ablation, LPN = laparoscopic partial nephrectomy.

## Data Availability

The data that have been used are confidential.
